# Gas Turbine Anomaly Detection under Time-Varying Operation Conditions Based on Spectra Alignment and Self-Adaptive Normalization

**DOI:** 10.3390/s24030941

**Published:** 2024-01-31

**Authors:** Dongyan Miao, Kun Feng, Yuan Xiao, Zhouzheng Li, Jinji Gao

**Affiliations:** State Key Laboratory of High-End Compressor and System Technology, Beijing University of Chemical Technology, Beijing 100029, China

**Keywords:** gas turbine, anomaly detection, frequency spectrum, neural network, time-varying operating conditions, normalization method

## Abstract

Gas turbine vibration data may exhibit considerable differences under time-varying conditions, which poses challenges for neural network anomaly detection. We first propose a framework for a gas turbine vibration frequency spectra process under time-varying operation conditions, assisting neural networks’ ability to capture weak information. The framework involves scaling spectra for aligning all frequency components related to rotational speed and normalizing frequency amplitude in a self-adaptive way. Degressive beta variational autoencoder is employed for learning spectra characteristics and anomaly detection, while a multi-category anomaly index is proposed to accommodate various operating conditions. Finally, a dataset of blade Foreign Object Damage (FOD) fault occurring under time-varying operating conditions was used to validate the framework and anomaly detection. The results demonstrate that the proposed method can effectively reduce the spectra differences under time-varying conditions, and also detect FOD fault during operation, which are challenging to identify using conventional methods.

## 1. Introduction

Gas turbines are highly expensive industrial equipment that find widespread use in numerous fields such as ship propulsion, gas transmission, and electric power generation due to their reliability as well as practicality [1,2]. Functioning within challenging environments marked by elevated temperatures, increased pressures, and high rotational speeds, components of gas turbines are susceptible to failures. n severe cases, these failures can even lead to catastrophic accidents [3]. Condition monitoring and real-time anomaly detection of gas turbines are important measures to improve equipment reliability, ensure operational safety, and reduce equipment maintenance costs [4].

Analyzing vibration data to detect equipment anomalies constitutes a widely employed approach, given that equipment failures often manifest prominently in vibration data. Numerous scholars have thus far applied anomaly detection methods based on vibration signals to diverse categories of rotating machinery, encompassing gas turbines [5,6,7,8]. This practice underscores the effectiveness of utilizing vibration data for identifying potential anomalies in machinery operation.

Anomaly detection can be regarded as a one-class classification (OCC) task, where the objective is to learn a single data category and identify whether new data belong to this category [9,10]. Performing anomaly detection without any fault samples available in the training set is indeed a form of one-class classification task [11]. As a machine learning approach, neural networks have been widely applied in the industry. Due to their data-driven nature, neural networks are capable of extracting features from monitoring data without prior information [12] and performing anomaly detection [13,14] on equipment. Autoencoder and its variants are often used for anomaly detection tasks due to their unsupervised nature [15,16]. Anomaly detection using neural networks with an encoder–decoder structure generally relies on reconstruction errors to determine the degree of abnormalities [17,18,19,20,21].

The direct application of raw vibration waveform data in training neural network models is challenging, as these waveforms do not distinctly reveal too much useful information. As a result, the adoption of feature engineering for the extraction of relevant features is a common choice. Indeed, neural networks possess robust feature extraction capabilities; many feature extraction tasks are also accomplished by neural networks [22,23]. Therefore, a potentially superior approach may involve inputting data into the neural network that encompass as much information as possible, allowing the network to autonomously discern and prioritize effective information. In such a scenario, the vibration spectrum emerges as a favorable option, as it maximally preserves the amplitude information of each frequency component and distinctly represents this information. Numerous studies have employed vibration spectra for neural network-based anomaly detection in rotating machinery [20,21,24].

However, given the high complexity of gas turbines, various factors impact the acquired vibration waveforms and corresponding spectra. Temperature fluctuations, the installation position and orientation of sensors, along with a substantial amount of mechanical noise are crucial considerations. These factors may introduce significant disturbances during the data analysis process. It is noteworthy that changes in the gas turbine speed have the most pronounced effect on the spectrum. Depending on the application scenario, gas turbines may need to operate under time-varying operating conditions, and the variations in rotational speed can significantly impact the distribution of the spectrum. In such a situation, when the fault severity is not substantial, fault features may be obscured by variations in rotational speed. The significance of components corresponding to the same frequency in vibration spectra at different rotational speeds varies. Neural networks are unable to effectively match frequency components in the spectrum that occupy different positions due to varying rotational speeds, significantly impairing the learning capability of the neural network. There have been some studies dedicated to anomaly detection in time-varying operating condition equipment [25,26,27,28]. However, most of these studies have focused on bearings or gearboxes. Due to the high difficulty of data acquisition, research targeting gas turbines is comparatively scarce. The complexity of gas turbines further exacerbates the difficulty of achieving this objective.

In order to solve these problems, this paper proposes a spectral data process framework, which aligns the spectrum under time-varying operating conditions according to the rotational speed. This ensures that frequency components related to the rotational speed are aligned to the same position in each spectrum. Consequently, during the training of neural networks, components reflecting identical frequency information can be input to the same neurons. Then, a self-adaptive global normalization method is proposed to mitigate the impact of high-amplitude frequency components on the capabilities of neural networks. The goal of the methods is to improve the learning ability of the neural network to the information in the vibration spectrum. To demonstrate the effectiveness of the approach under variable operating conditions, a dataset from a real gas turbine with a Foreign Object Damage (FOD) [29] fault is utilized for validation. The main contributions of this paper are as follows:

- To address the issue of poor performance when using frequency spectra to train neural networks, a spectra alignment method is proposed. This method aligns the corresponding frequency components related to the rotational frequency of the spectrum to the same positions, making it compatible with neural networks.
- A self-adaptive global normalization method suitable for vibration spectra is proposed, which enhances features of weak components while preserving the distinctions of the importance of frequency components with different amplitudes. This approach enables neural networks to better learn information from the spectra.
- An entire abnormal detection framework for gas turbines was established with a more suitable anomaly index for time-varying operating conditions. And the effectiveness of the proposed methods was validated using a real gas turbine dataset.

The remainder of this article is organized as follows. The second part describes the spectra alignment method and the self-adaptive normalization method. The complete anomaly detection process used in the experiment is also presented. In the third part, the proposed method is validated with a real gas turbine dataset. Conclusions are presented in the fourth part.

## 2. Proposed Method

### 2.1. Spectra Alignment

Spectrum analysis is one of the most commonly employed methods in the field of fault diagnosis. The spectrum is obtained by the Fourier transform of the vibration waveform. In practical applications, the vibration waveform data that can be collected are discrete. Therefore, the Fast Fourier Transform (FFT) is commonly employed to compute the spectrum:

(1) $$S\left(\omega \right)=\mathrm{FFT}\left(s\left(N\right)\right)$$

where *S*(ω) represents the spectrum, *s*(*N*) represents the original vibration waveform, *N* represents the number of sampling points in the vibration waveform, and the length of the spectrum is consistent with it. Due to the symmetry of the spectrum, typically only the first half is considered in practice. Therefore, the effective length of the spectrum used is n=N/2. The vibration spectrum provides a clear representation of the signal in the frequency domain, facilitating a more effective visualization of the amplitude information of different frequency components. The spectrum enhances the identification and understanding of anomalies in vibration signals, playing a crucial role in frequency domain analysis and fault diagnosis.

The utilization of spectral data for training neural networks is a common practice. For neural networks, inputs may comprise either features derived from data or the complete dataset itself. Feature data comprise selected critical information, reflecting key object characteristics, thereby facilitating enhanced learning capabilities for neural networks. When a manually extracted feature is unable to meet the requirements, it is possible to input all the information into the neural network and let the neural network extract the effective information. Spectra can be regarded as a kind of whole data, and some researchers have utilized one-dimensional convolutional layers to process spectra data in a manner similar to image processing techniques [30]. On the other hand, it is important to acknowledge that there are differences between spectra and images. One of the rationales supporting the suitability of convolutional neural networks (CNNs) for image data processing lies in their capability to adeptly acquire spatial information in images. In other words, CNNs are capable of capturing the correlations between adjacent pixels, thereby acquiring knowledge about the spatial relationships within the image [31]. However, there is generally no significant correlation between adjacent spectral lines in the frequency domain. Hence, it is not optimal to use a convolutional neural network to process the spectra like pictures but to treat each spectral line of the spectrum as an independent feature.

**Figure 1:**
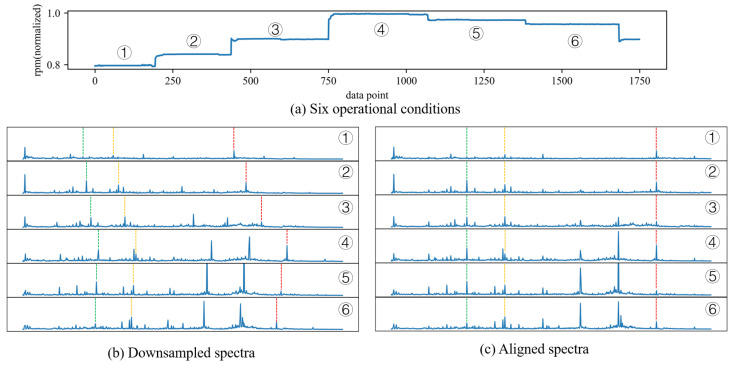
Demonstration of spectra alignment using gas turbine vibration spectra under six operational conditions.

However, under variable operating conditions, directly using the spectral data as features for training a neural network consisting of fully connected layers is also not an ideal choice. For instance, in Figure 1b, frequency components with the same meaning are denoted by lines of the same color, yet their positions within the spectrum differ. Hence, if the spectrum is directly fed into a neural network, the features entering the same neural unit will not be consistent with each change in rotational speed. This makes it difficult for the neural network to extract meaningful information from the data. Hence, it is crucial to align the frequency components with the same significance in spectra to the same positions before feeding them into the neural network. This alignment process ensures that the network can effectively capture the relevant information and improve accuracy and interpretability. The results obtained from this operation are similar to the order spectrum. However, order spectra are primarily used for cases where the rotational speed is continuously changing, and they require higher demands on data acquisition. It is necessary to have key phase signals with the same sampling frequency as the vibration signal. However, in practical gas turbine monitoring, achieving this is challenging. In this study, the acquired vibration data comprise not an entirely continuous waveform but rather numerous short waveform segments, each with a brief data collection period. The rotational speed of the gas turbine transitions between several stable values instead of exhibiting continuous changes, and each waveform segment corresponds to a nearly constant rotational speed. Consequently, order spectrum analysis is not applicable to such data. The proposed spectra alignment method in this paper is more easily implementable and preserves the meaningfulness of amplitude information, better meeting the requirements of neural networks for data processing.

To align the spectra, it is necessary to subject the spectra to varying degrees of compression, contingent upon the rotational speeds. The compression rate *r* of the spectrum is defined by the following equation:

(2) $$r=\frac{n}{m}$$

where *n* represents the length of the original spectrum, and *m* represents the length of the aligned spectrum. The maximum compression rate, denoted as γ, is defined based on the spectrum with the highest rotational speed in the training set. To ensure alignment of the compressed spectra, *r* for other spectra can be calculated using the following formula:

(3) $$r=\frac{\gamma \times rpm}{rpm_{\max}}$$

where rpm is the rotational speed corresponding to the spectrum, and $rpm_{\max}$ is the maximum rotational speed of the training set. The maximum compression rate γ is decided according to the actual situation, and its value should be at least larger than the ratio of the largest rotational speed to the smallest rotational speed in the training set; otherwise, some of the spectrum compression rates will be less than 1. A larger γ can be chosen to reduce the data length when the spectral data are long, which can reduce the number of neural network parameters, similar to pooling operations. Define $m_{\min}$ as the length of the compressed spectrum corresponding to the maximum compression rate. In order to ensure the same length of the compressed spectra, only the first $m_{\min}$ data points are retained in each spectrum. In practice, the test set may have a higher rotational speed than the training set, so a figure smaller than $m_{\min}$ can be chosen as the length of the retained data.

**Figure 2:**
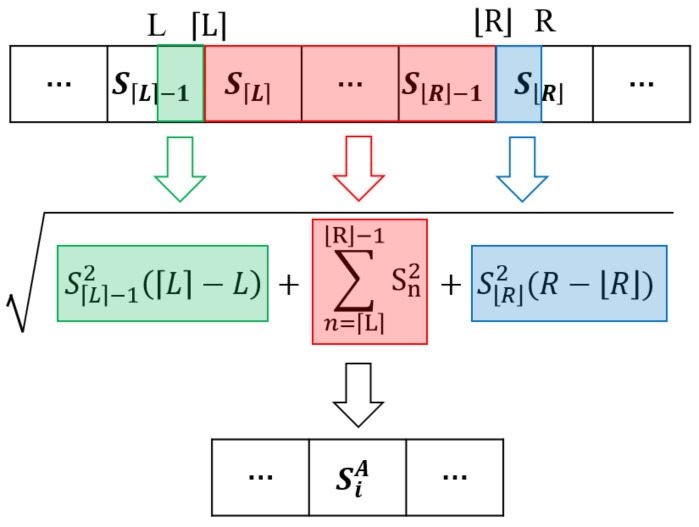
Demonstration of Equation (6).

The compression operation of the spectrum is to merge *r* consecutive data points into one dataset with the spectrum energy constant. It is merged by obtaining the sum of squares of these data and then taking the root of the sum. The *i*-th data of the merged spectrum is calculated by the following equations:

(4) L=(i−1)r

(5) R=ir

(6) $$S_{i}^{A}=\sqrt{\sum_{n=\left\lceil L\right\rceil }^{\left\lfloor R\right\rfloor -1}S_{n}^{2}+S_{\left\lceil L\right\rceil -1}^{2}\left(\left\lceil L\right\rceil -L\right)+S_{\left\lfloor R\right\rfloor }^{2}\left(R-\left\lfloor R\right\rfloor \right)}$$

where *L* and *R* are the left side and right side of the merging range, $\left\lfloor •\right\rfloor$ denotes rounding down to the nearest integer, $\left\lceil •\right\rceil$ denotes rounding up to the nearest integer, $S_{i}^{A}$ is the *i*-th term of the aligned spectrum, $S_{n}$ is the *n*-th term of the original spectrum. The *i*-th data point of the merged spectrum is obtained by combining *L*-th to *R*-th data points of the original spectrum, but since *r* is not an integer, the head and tail data points need to be sliced during the merging. The computation process of Formula (6) can be more readily comprehended through Figure 2. A special case is that when *L* is equal to 0, $S_{\left\lceil L\right\rceil -1}^{2}$ cannot be calculated—it should be set to zero—and when *R* is equal to the length of the spectrum, $S_{\left\lfloor R\right\rfloor }^{2}$ also should be set to zero. This adjustment ensures the proper calculation of the formula.

Figure 1 demonstrates the effect of spectra alignment under six different operating conditions (①–⑥). Figure 1a shows the six working conditions of the spectra, Figure 1b shows the result of reducing using a ratio of 10:1, where the square root of the quadratic sum of the 10 data points is taken in order to be consistent with the operation of spectra alignment, and Figure 1c shows the result of spectra alignment with the parameter γ equal to 10. Three frequency components significantly correlated with the rotational frequency are marked with dashed lines of three colors in Figure 1, and since each spectrum corresponds to a different rotational speed, the corresponding frequency components in Figure 1b all have different positions which are related to the rotational speed, while in Figure 1c, the corresponding frequency components are all at the same position.

### 2.2. Self-Adaptive Global Normalization

Before training a neural network, the data are generally normalized to avoid gradient explosion and to speed up the training process of the network by adjusting the data range. The common normalization method is a linear variation of the original data to map the data to the range [0,1]. Each spectrum can be viewed as a one-dimensional array. There are three normalization methods that can be chosen for such data:

1. Feature normalization: Each corresponding data point across various spectra is normalized using the same parameters. This means that the normalization is performed independently for each position across all spectra.
2. Instance normalization: Each spectrum is normalized using the same parameters. This normalization is applied separately to each spectrum, regardless of the position of the data points.
3. Global normalization: All the data points in all the spectra are normalized using the same parameters. The normalization is performed collectively on all the spectra, treating them as a single set of data.

As mentioned in the previous section, each data point in the spectrum can be considered as a separate feature. Therefore, it is evident that feature normalization is a suitable normalization method for spectra. However, in practice, the best result is achieved by using global normalization and employing a self-adaptive nonlinear normalization method.

The main idea of self-adaptive normalization is to linearly transform the majority of the main data points to the range [0,1] and apply a nonlinear transformation to extreme outliers. This method utilizes a nonlinear piecewise function to map all the data points to the range of (−1, 2). The function is defined as follows:

(7) $$x_{\mathrm{norm}}=\left\{\begin{array}{c} -e^{\left(1-\frac{x-k_{1}}{k_{2}-k_{1}}\right)}+2,\;x>k_{2}\\ \frac{x-k_{1}}{k_{2}-k_{1}},\;k_{1}\leq x\leq k_{2}\\ e^{\left(\frac{x-k_{1}}{k_{2}-k_{1}}\right)}-1,\;x<k_{1} \end{array}\right.$$

where *x* is the original data, $x_{\mathrm{norm}}$ is the normalized data, $k_{1}$ and $k_{2}$ are the two parameters of normalization, which act similarly to the maximum and minimum values in linear normalization, and their values are determined by the distribution of the data. The upper bound $t_{1}$ for $k_{1}$ and the lower bound $t_{1}$ for $k_{1}$ are first determined using the quadrature distance method:

(8) $$IQR=Q_{3}-Q_{1}$$

(9) $$t_{1}=Q_{1}-1.5\times IQR$$

(10) $$t_{2}=Q_{3}+1.5\times IQR$$

where $Q_{1}$ represents the first quartile, $Q_{3}$ represents the third quartile, and IQR represents the interquartile range. To prevent the clustering of data from being divided by $k_{1}$ or $k_{2}$, it is necessary to determine the suitable splitting points based on the density of the data. Here is the process to determine the splitting points:

1. Sort the data in ascending order.
2. Compute the difference between two adjacent data points.
3. Compute the average of these differences and multiply it by 10 to obtain the threshold value.
4. Identify all data points whose difference values exceed the threshold. These points are considered as potential splitting points.
5. Find $k_{1}$, which is the first splitting point that is less than or equal to $t_{1}$.
6. Find $k_{2}$, which is the left neighbor of the first splitting point that is greater than or equal to $t_{2}$.

For the sake of facilitating comprehension of the computational procedure, Algorithm 1 provides the pseudocode for this algorithm.
**Algorithm 1** Find Normalization Parameters k1 and k2**Require:**  *data* (array of numeric values), *t*1 (numeric value), *t*2 (numeric value) **Ensure:**  *k*1, *k*2 (numeric values)
  1:Sort the data in ascending order.  2:Initialize an empty array difference.  3:**for** i←0 to len(data)−2 **do**  4:    difference[i]←data[i+1]−data[i]  5:**end for**  6:threshold←mean(difference)×10  7:index←where(difference>threshold)  8:**for** i←0 to len(index)−2 **do**  9:    **if** data[index[i]]≤t1 **and** t1<data[index[i+1]] **then**10:        k1←data[index[i]]11:    **end if**12:    **if** data[index[i]]<t2 **and** t2≤data[index[i+1]] **then**13:        k2←data[index[i+1]+1]14:    **end if**15:**end for**


**Figure 3:**
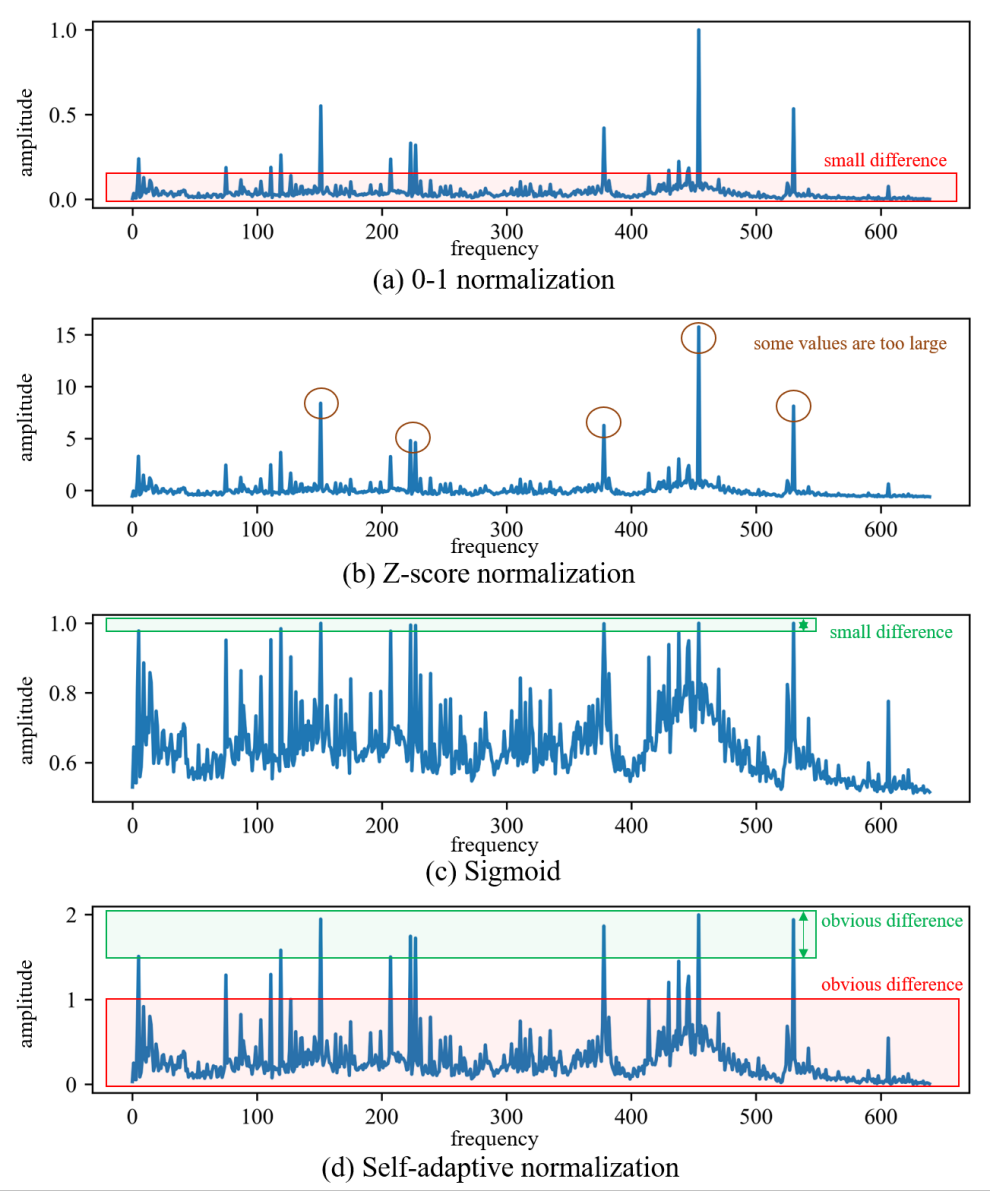
Comparison of proposed self-adaptive normalization and some common normalization methods.

This approach helps ensure that data points that are closely clustered together are not split by the selected splitting points, allowing for a more appropriate determination of the splitting points based on the density of the data. The difference between proposed self-adaptive normalization and some common normalization methods is visualized in Figure 3. The use of 0–1 normalization methods can diminish the differences in low-amplitude frequency components, making it difficult for neural networks to learn meaningful information from them, especially when certain frequency components have extremely high amplitudes. The data range after Z-score normalization may be extensive, and excessively large numerical values can induce instability during neural network training, thereby compromising the learning capacity of the neural network. The Sigmoid function is a non-linear normalization function commonly employed as an activation function in neural networks. It lacks the ability to adjust its parameters based on the target data, rendering its effectiveness relatively unstable. As illustrated in Figure 3c, the differences in high-amplitude data are excessively diminished. Self-adaptive normalization enhances the distinctiveness of low-amplitude components of different spectra while preserving the information of high-amplitude components. This allows neural networks to capture more information from spectra. Choosing overall normalization along with the self-adaptive normalization method can reduce the overall impact of the very high-amplitude spectral components of the spectra with high rotational speed. Instance normalization can achieve the same benefits by using the self-adaptive method, as shown in Figure 3d. However, since feature normalization employs different parameters for different frequency components, the self-adaptive method does not offer significant improvements in this case. The reason for choosing adaptive global normalization is that it can amplify the information of low-amplitude components while preserving the importance differences among different components. In general, frequency components with higher amplitudes tend to carry more significance in the spectrum. By using adaptive global normalization, the neural network can appropriately scale the amplitudes of all components, thereby ensuring the preservation of significance differences among various frequency components. But feature normalization does not consider the varying importance levels of different frequency components based on their amplitudes.

### 2.3. Anomaly Detection Process

**Figure 4:**
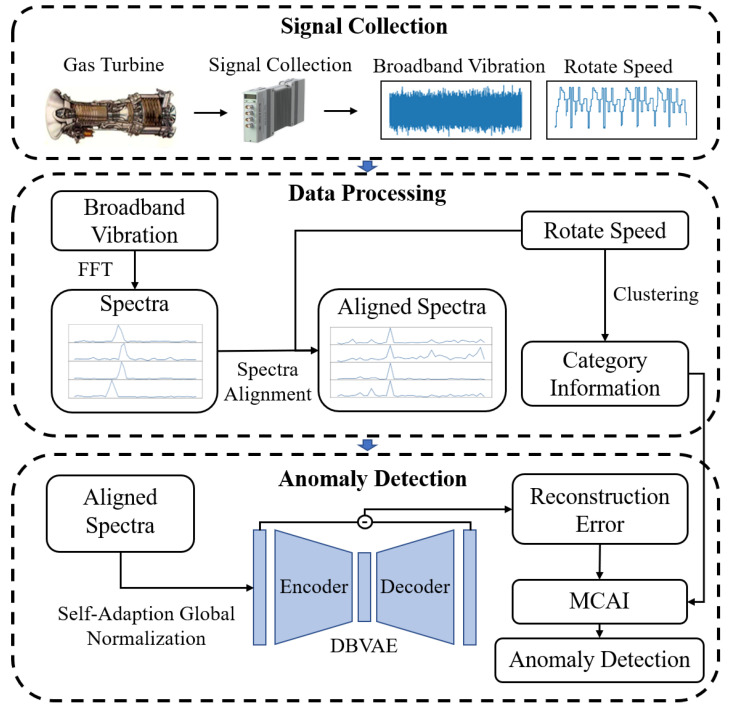
Flowchart of the proposed gas turbine anomaly detection method process.

Figure 4 shows a complete gas turbine anomaly detection process, including data acquisition, spectra alignment, data normalization, training neural networks, and constructing anomaly indicators. The neural network adopts the β-VAE using a progressive training method, which is called Degressive Beta Variational Autoencoder (DBVAE) in this paper in order to differentiate from the original β-VAE. The anomaly indicator uses the reconstruction error adjusted according to the working condition information, which can achieve better performance on data under time-varying operation conditions.

The β-VAE was initially proposed and applied in the field of computer vision [32], and then its disentangling ability was discussed [33]. In this study, we adopted the model architecture of β-VAE and employed a similar progressive training approach. Compared to conventional autoencoder (AE), this model has higher robustness. The loss function of the ordinary β-VAE is as follows:

(11) $$L\left(\theta ,\phi ;x,z,\beta \right)=E{q_{\phi }}_{\left(z|x\right)}\left[\log p_{\theta }\left(x|z\right)\right]-\beta D_{KL}\left(q_{\phi }\left(z|x\right)\left|\right|p\left(z\right)\right)$$

where the first item in the formula is reconstruction error and the second item is Kullback–Leibler loss (KL loss), which is the KL divergence between the distribution of the latent variable and the Gaussian distribution. In β-VAE, the latent variables are not learned directly as values, but rather as distributions. The KL divergence between the distribution used for sampling the latent variables and the Gaussian distribution represents the amount of information contained in those latent variables. A higher KL divergence indicates a greater amount of information captured by the latent variables. The KL loss in β-VAE introduces an information bottleneck in the model. The parameter β controls the strength of this bottleneck. A higher value of β imposes a stronger constraint on the bottleneck, resulting in a more compact representation of the latent variables. Conversely, a smaller value of β relaxes the constraint and allows for more expressive latent variables.

The progressive training method for DBVAE starts with a relatively large initial value of β during the early stages of training to encourage disentanglement of the latent variables. As the training progresses, the value of β is gradually decreased, allowing the model to improve the reconstruction accuracy of the input data. The strategy for adjusting the β value is as follows:

(12) $$\beta =b\times 0.5^{iter\times d}$$

where iter represents the number of training iterations, and *b* and *d* are two parameters that are used to control the initial value and the rate of decrease for β, respectively. This training strategy allows the latent variables of the model to be activated one by one, rather than increasing the information content of all latent variables simultaneously. This prioritization of learning important features and ignoring minor information helps make the learned features more stable and reduces interference, thereby improving the model’s robustness.

Anomaly detection tasks using models with an encoder–decoder structure generally use the mean square error (MSE) of the model input data and output data to determine the degree of anomalies. However, in this task, the vibration amplitude varies with the rotational speed, and the MSE values can differ between different operating conditions. Generally, data with larger amplitudes correspond to larger MSE values. In order to minimize the interference caused by this phenomenon, we propose a multi-category anomaly index (MCAI), which is obtained by adjusting the MSE values according to the categories. MCAI is calculated by the following equation:

(13) $$MCAI_{i}=\frac{MSE_{i}-\mu_{n}}{\sigma_{n}}-c$$

where *i* is the index of data, *n* is the category of the *i*-th data, $\mu_{n}$ and $\sigma_{n}$ are, respectively, the mean and standard deviation of all MSE in category *n* from the validation set. *c* is the minimum MCAI value of the training set and validation set, which is used to adjust the MCAI value to a positive scale.

While rotational speed is not the sole indicator of the operating condition of the engine, manual classification based on actual operating conditions would be the best choice. However, in practical industrial applications, it may be difficult to manually classify data based on operating conditions. In such cases, the clustering method is a good alternative approach. Since the rotational speed comprises relatively simple, one-dimensional data, the clustering method directly adopts the classical k-means clustering algorithm [34], which has a good effect and fast convergence. The number of clusters in the k-means algorithm can be determined by comparing the silhouette coefficient [35] of different clustering results.

## 3. Experimental Results and Analysis

### 3.1. Dataset Description

**Figure 5:**
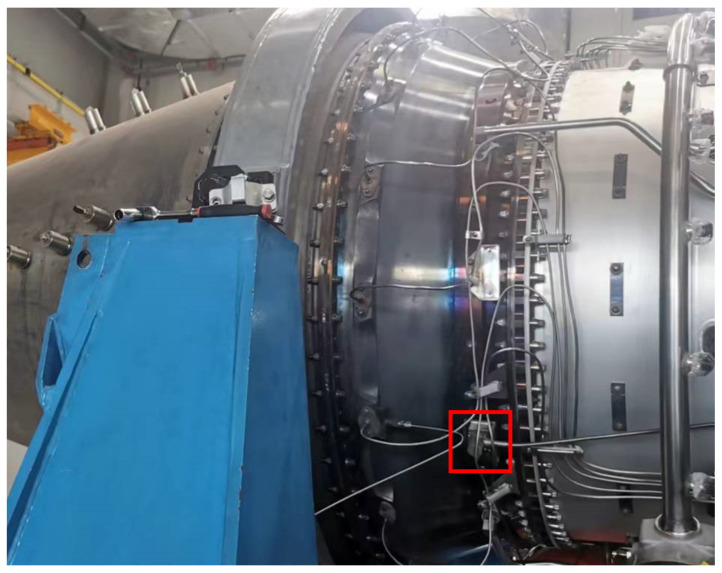
The mounting position of the acceleration vibration sensor is indicated by the red box.

**Figure 6:**
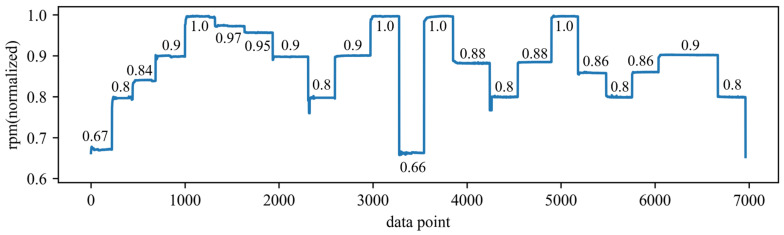
The change in low-pressure air compressor rotational speed in the first test run.

The effectiveness of the proposed method is validated using a set of vibration data collected from a real gas turbine. The signal is captured using a data acquisition unit with an acceleration sensor installed in the horizontal of the inlet casing as shown in Figure 5. The data were sampled at a frequency of 51.2 kHz with a sampling interval of 3 s. Each sample consists of a vibration signal of 0.32 s, resulting in a sample length of 16,384. The dataset comprises eight test runs. Figure 6 illustrates the variations in the low-pressure compressor’s rotational speed during the first test run, with the subsequent seven test runs exhibiting similar patterns. The speed value in Figure 6 is normalized by dividing it by the maximum speed value, and it is only used to show the trend of change in working conditions. During the fifth operational run, an incident of Foreign Object Damage (FOD) occurred due to the ingress of small metal fragments into the low-pressure compressor. The suddenness of FOD makes it unpredictable. The magnitude of this failure was so minor that it did not even affect the normal operation of the combustion turbine, and, due to the low level of the failure, resulted in its features being masked by the changing operating conditions. The complexity inherent in these factors renders the detection of such failures exceptionally challenging. The dataset initially contained 64,977 samples, and after filtering out the data during shutdown periods, a total of 55,082 usable samples remained. Among these samples, there are 33,963 samples representing healthy operating conditions and 21,119 samples representing abnormal operating conditions. In the subsequent experiments, the first test run dataset consisting of 6963 samples will be used as the training set, the second test run dataset consisting of 6983 samples will be used as the validation set for model adjustment and anomaly indicators, and the remaining data will be used as the test set.

### 3.2. Validation and Comparison

**Figure 7:**
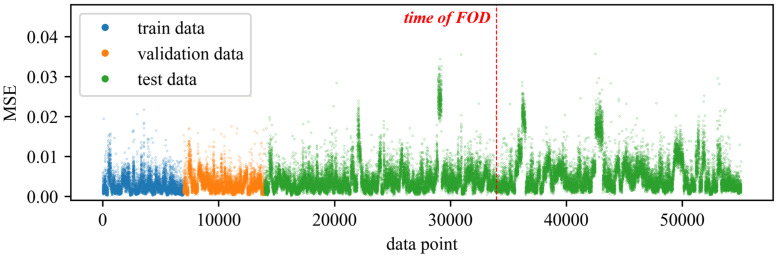
The results of using the DBVAE model for anomaly detection with traditional time-domain and frequency-domain features.

**Figure 8:**
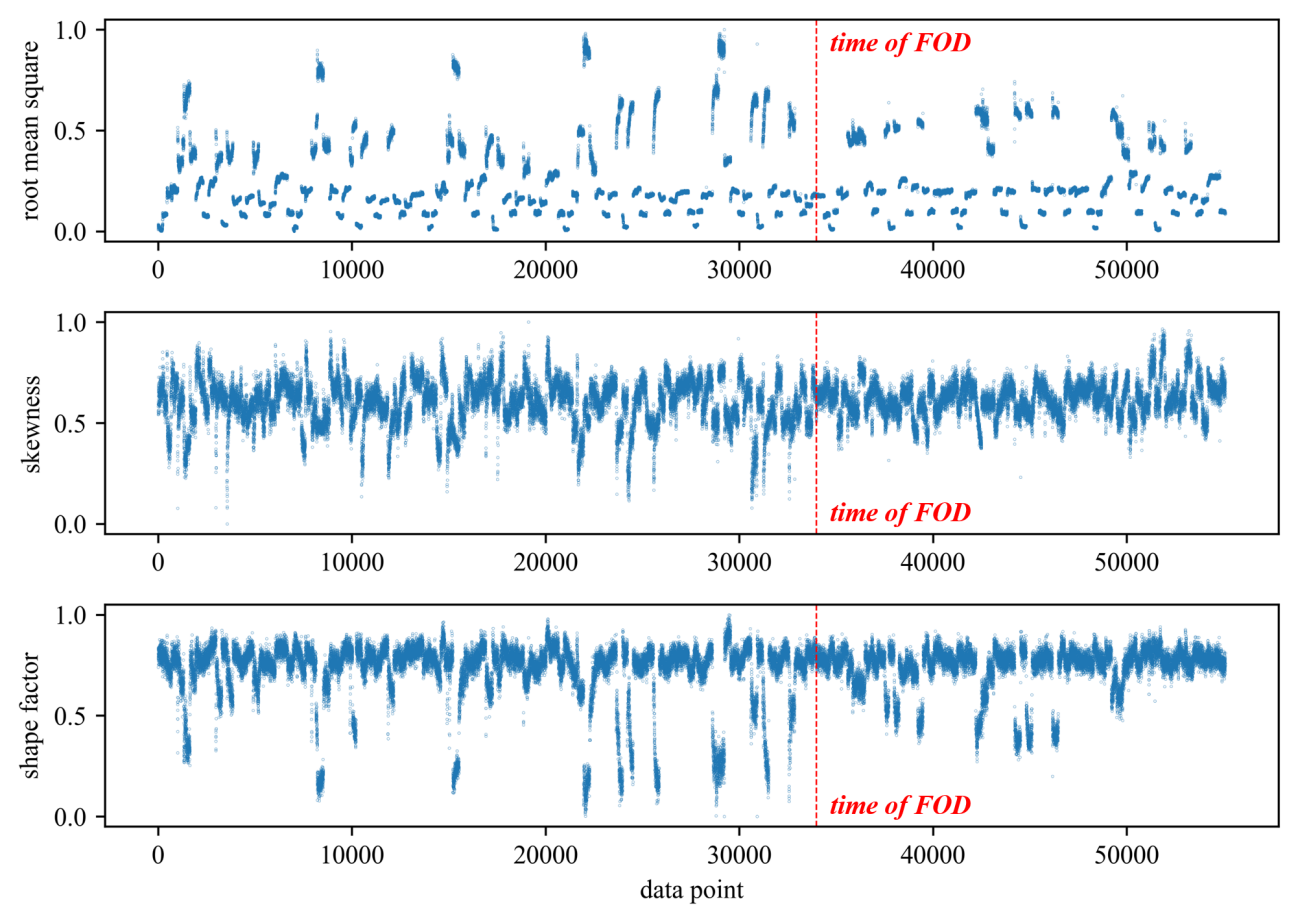
Visualization of three prominent features in traditional features: root mean square, skewness, and shape factor.

To validate the effectiveness and superiority of the proposed method, a comparison was made with traditional feature extraction methods for anomaly detection. Specifically, 20 time-domain and frequency-domain features were extracted using traditional feature extraction techniques. The extracted time-domain features include mean, absolute mean, variance, standard deviation, peak, root mean square, root mean square amplitude, kurtosis, skewness, crest factor, peak-to-peak, average energy, clearance factor, waveform factor, and impulse factor. The extracted frequency-domain features include spectral centroid, mean frequency, root mean square frequency, standard deviation frequency, and spectral entropy. The extracted time-domain and frequency-domain features, along with the rotational speed, totaling 21 features, were used to train the DBVAE network. All neural network models in this paper are constructed using TensorFlow 2.6 and trained utilizing NVIDIA GeForce GTX 1050 Ti. The Adam optimizer was employed with a learning rate of 0.001. The initial beta value *b* was set to 0.01, and the β decay rate d was set to 0.001. The network was configured with eight latent variables, and both the encoder and decoder consisted of two hidden layers with 32 neurons each. The obtained results of anomaly detection are shown in Figure 7. The results show that the anomaly index seems increase slightly after the occurrence of the fault compared to before. However, the differences are not significant, and there are some instances where higher anomaly values may be attributed to operating conditions outside the range of the training set. As a result, it is difficult to determine the exact time of the fault occurrence or even confirm whether a fault has occurred in the engine. Figure 8 shows some of the extracted features, and the features are greatly affected by the change in rotational speed. In the figure, the last three tries have some distributional differences from the previous data. Although the distribution of the features before and after the failure is different under specific operating conditions, especially under high operating conditions, the overall distribution of the features after the failure is still within the range of the pre-failure distribution, which is why it is difficult to detect the anomaly.

When employing full-spectrum data for anomaly detection, the neural network’s parameter count is reduced by removing high-frequency components that lack effective information from the obtained spectra. Consequently, the resultant length of each spectrum is 6400. The spectra are then aligned with the parameter γ set to a value of 10, and the final length of the data samples used for training the neural network is reduced to 640. In order to obtain data of the same length, the data without spectra alignment were reduced in a 10:1 ratio using the method of calculating the square root of the sum of squares for every 10 data points. It is noteworthy that the computational time for the individual spectrum with this process is approximately 3.09 ms, while the time for spectrum alignment is about 3.49 ms, indicating little difference between the two methods. However, as the data scale increases, the computation time for spectrum alignment grows linearly due to the challenges in vectorized operations, highlighting its computational disadvantage. Nevertheless, in real-time anomaly detection scenarios, where processing large volumes of data simultaneously is rarely encountered, this disadvantage is practically inconsequential.

**Figure 9:**
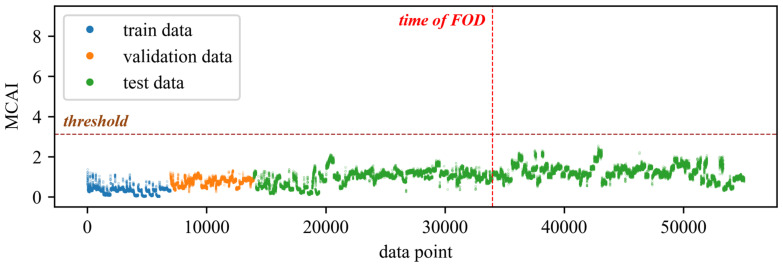
The results of using the DBVAE model for anomaly detection without spectra alignment.

**Figure 10:**
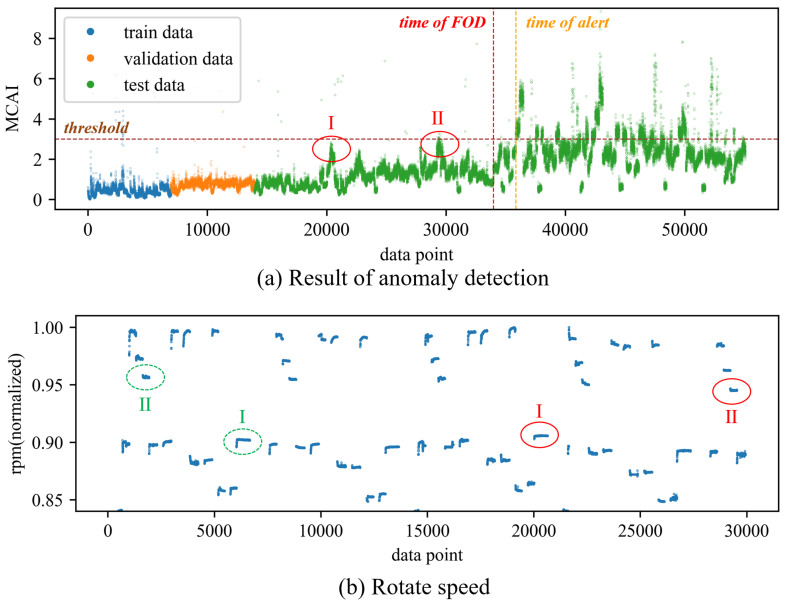
The results of using the DBVAE model for anomaly detection with spectra alignment and rotational speeds corresponding to partial data.

**Figure 11:**
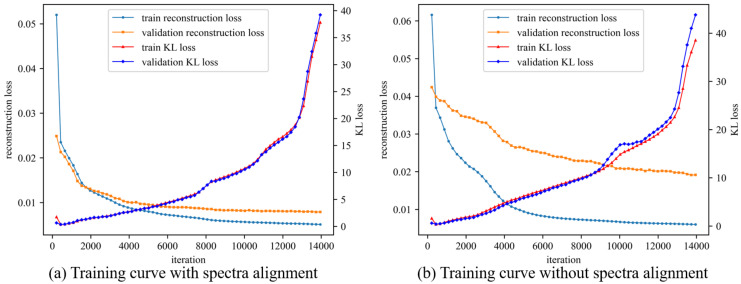
Training curves of the DBVAE models for anomaly detection with and without spectra alignment.

In this experiment, the model is configured with 32 latent variables, and both the encoder and decoder have two hidden layers with 64 neurons each. The optimizer used is Adam with a learning rate of 0.001. The parameter *b* is set to 0.01, and the parameter *d* is set to 0.001. Figure 9 and Figure 10 show the anomaly detection results obtained without and with spectra alignment, respectively. Additionally, Figure 11 shows the training curves of the two models. The alarm threshold is determined based on the validation set and is set to three times the 95th percentile of the anomaly index in the validation set. In Figure 9, on the whole, it can be observed that the anomaly index shows a slight increase after the occurrence of the fault. However, it is difficult to determine the occurrence of the fault from the graph. In Figure 10a, the anomaly occurs at the 33,964th data point, and the alarm is triggered at the 35,845th data point, which is approximately 1.5 h after the occurrence of the fault. The false alarm rate is about 0.15%. Indeed, given the relatively mild severity of the fault, its impact on the engine’s operation was minimal. The detection of the fault occurred during an internal bore inspection conducted after the engine had been in continuous operation for a certain duration. In comparison to the aforementioned scenarios, the proposed method was able to detect the anomaly earlier. There are two main reasons for the delay in the alarm after the occurrence of the fault. Firstly, the fault had a relatively low severity, and some operating conditions, especially low rotational speed, did not exhibit significant anomalies in the vibration data. Secondly, the limited training data coverage contributed to the issue. The training data covered a narrow range of operating rotational speeds, and data points with speeds outside the training range resulted in higher anomaly indexes. To reduce false alarms, a higher threshold was set for the alarm, leading to a delay in detection. The difference in rotational speed between the data points with high anomaly scores and the data in the training set is shown in Figure 10b. The two red circles on the right correspond to the high-anomaly regions in Figure 10a. The two green circles on the left represent the rotational speed of the corresponding operating conditions in the training set. With a larger training dataset that covers a wider range of rotational speeds, the false alarm rate of this method can be further reduced. Moreover, it would enable faster detection and alarm for faults with higher levels of anomaly.

To quantitatively evaluate the efficacy of both spectra alignment and self-adaptive normalization, the AUC (Area Under Curve) value is employed as the comparative metric. The AUC value is defined as the area beneath the Receiver Operating Characteristic (ROC) curve. The ROC curve’s horizontal axis represents the false positive rate, while the vertical axis represents the true positive rate. The AUC value ranges from 0 to 1, with higher values signifying superior performance in distinguishing between healthy and anomalous data. An AUC value of 1 indicates an ideal classifier capable of completely segregating healthy and anomalous data, while an AUC value of 0.5 implies performance equivalent to random guessing.

In Table 1, clustering MCAI is obtained by adjusting the MSE by using the clustering result of k-means; manual MCAI is obtained by adjusting the MSE based on the manual classification of the operating conditions. Manual MCAI shows better performance than clustering MCAI, but the difference is minimal. This implies that in scenarios where the manual classification of operating conditions poses challenges, employing clustering methods as an alternative can yield comparable outcomes. Comparing the results with and without spectra alignment, it can be observed that spectra alignment greatly improves the effectiveness of anomaly detection in all cases. Comparing the six normalization methods after spectra alignment, it can be seen that self-adaptive global normalization performs the best when using the effective MCAI. Self-adaptive instance normalization is the next best option, while global normalization performs the worst. Table 1 suggests that the self-adaptive normalization method significantly improves the global normalization and also provides some improvement over instance normalization, while feature normalization was almost unaffected. Compared to feature normalization methods, self-adaptive global normalization, while aiming to enhance the neural network’s ability to capture information from low-amplitude components to the greatest extent possible, preserves the amplitude differences between various frequency components. Although low-amplitude components may also contain valuable information, it is evident that the significance of high-amplitude components is more pronounced. The retention of this differential information is why self-adaptive global normalization achieves optimal effectiveness with the assistance of spectra alignment. However, despite the remarkable effectiveness of self-adaptive normalization, its computational speed is slightly slower compared to the conventional 0–1 normalization method, primarily due to the use of piecewise functions in the data transformation process. In the processing of one hundred thousand data points, self-adaptive normalization takes 0.0185 s, while conventional 0–1 normalization takes 0.0184 s. However, with a dataset size in the order of ten million, the respective times are 2.84 s for self-adaptive normalization and 1.60 s for conventional 0–1 normalization. Therefore, in cases where the dataset is relatively small or when prioritizing method effectiveness, self-adaptive normalization can be employed. However, in situations where the dataset is large and there is a higher demand for computational efficiency, feature normalization should be preferred.

To compare the effect of spectra alignment on the enhancement of fully connected and one-dimensional convolutional layers, DBVAE as well as three other models of classical encoder–decoder structures were used for validation, and the results are shown in Table 2. All models have the same optimizer settings and latent variable count as the DBVAE model discussed in the previous section on processing spectral data. The configuration of hidden layers in the fully connected networks is consistent with the previous subsection. The configuration of encoders and decoders in the networks with convolutional layers is described in Table 3. The β value for β-VAE models is set to 0.01, and the β variation parameter for DBVAE models is the same as mentioned in the previous section. Overall, the effects of using spectra alignment are all significantly better than not using spectra alignment, and the enhancement effect of spectra alignment on the fully connected layer is better than its enhancement effect on the convolutional layer, with the best effect of DBVAE using the fully connected layer. This is because, without alignment, the convolutional layers are able to learn spatial information from the spectrum, resulting in better performance. On the other hand, after alignment, the data are treated as separate features, and the spatial information becomes less effective, leading to the convolutional layers performing worse than the fully connected layers.

**Figure 12:**
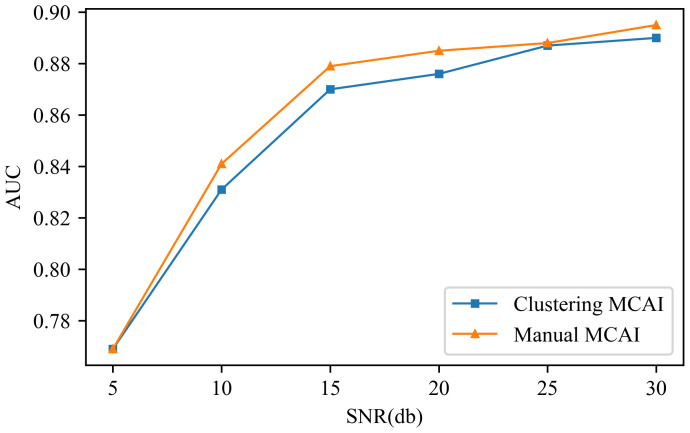
The variation in AUC values with the intensity of added noise.

Due to the utilization of experimentally acquired data in the study, it inherently contains a certain level of noise interference. To assess the proposed method’s resilience to noise, additional noise of varying intensities was deliberately introduced into the dataset. In this experiment, spectra alignment and global self-adaptive normalization were employed, while the parameters of the DBVAE network were configured with the same settings as described earlier. Figure 12 illustrates the variation curve of AUC values under different signal-to-noise ratios (SNRs), where a higher SNR corresponds to lower added noise energy. It can be observed that the AUC value experiences a substantial decrease when the SNR is 10. However, for SNR values greater than 15, the performance degradation of the proposed method becomes less pronounced.

## 4. Conclusions

In order to realize the anomaly detection of gas turbines in time-varying operating conditions, this paper proposes a data processing framework for improving the performance of neural networks. By utilizing spectra alignment, the learning ability of the neural network on the spectrum data can be effectively improved. Furthermore, the application of self-adaptive global normalization preserves the characteristics of prominent components in the spectrum, simultaneously emphasizing the features of weak components, thereby augmenting the learning efficacy of the neural network. The effectiveness of the proposed method was verified by a set of gas turbine FOD fault data collected under time-varying operating conditions. The method proposed in this paper solves the problem of the ineffectiveness of using neural network models to analyze spectral data obtained under different rotational speeds. In the experiment, an AUC value of 0.903 was achieved. Moreover, this method can be extended to other rotating equipment for anomaly detection tasks and fault diagnosis tasks under time-varying operating conditions.

The effectiveness of the method relies on having enough data that include a wide range of operating conditions. If the operating conditions to be detected deviate significantly from the training data, even in the absence of faults, the anomaly indicator may exhibit higher values, thereby impacting the accuracy of the anomaly detection results. Therefore, improving the model’s generalization capability to perform well on operating conditions beyond the training set is a valuable research goal. In addition, it is evident that spectra alignment exhibits favorable performance primarily in scenarios involving variations in rotational speed, whereas normalization methods are generally applicable across a broader range of situations.

## Figures and Tables

**Table 1:** AUC values obtained from anomaly detection using DBVAE model.

Spectra Alignment	Normalization Method	MSE	Clustering MCAI	Manual MCAI
×	Instance	0.624	0.629	0.627
	Self-adaptive Instance	0.526	0.536	0.549
	Feature	0.629	0.668	0.675
	Self-adaptive Feature	0.630	0.692	0.667
	Global	0.521	0.504	0.516
	Self-adaptive Global	0.614	0.652	0.639
✓	Instance	0.738	0.773	0.769
	Self-adaptive Instance	0.719	0.807	0.810
	Feature	**0.824**	0.886	0.887
	Self-adaptive Feature	0.824	0.886	0.890
	Global	0.618	0.726	0.743
	Self-adaptive Global	0.782	**0.898**	**0.903**

**Table 2:** AUC values obtained for abnormality detection using 8 models.

Spectra Alignment	Fully Connected Layer					Convolutional Layer			
	**VAE**	***β*-VAE**	**AE**	**DBVAE**		**VAE**	***β*-VAE**	**AE**	**DBVAE**
×	0.633	0.632	0.603	0.652		0.633	0.649	0.665	**0.684**
✓	0.835	0.872	0.887	**0.898**		0.832	0.868	0.882	0.879

**Table 3:** Setting of hidden layers of encoders and decoders using convolutional layer.

Encoder			Decoder		
**Layer**	**Kernel Size**	**Stride**	**Layer**	**Kernel Size**	**Stride**
Conv1D	7×7×16	2	Conv1DTranspose	3×3×32	2
MaxPool1D	2×2	2	Conv1DTranspose	3×3×16	2
Conv1D	3×3×16	2	UpSampling1D	\	2
Conv1D	3×3×32	2	Conv1DTranspose	7×7×1	2

## Data Availability

Data available on request due to restrictions.

## References

[1] Fentaye A.D., Baheta A.T., Gilani S.I., Kyprianidis K.G. (2019). A Review on Gas Turbine Gas-Path Diagnostics: State-of-the-Art Methods, Challenges and Opportunities. Aerospace.

[2] Miao D., Liu B., Li Z., Li G., Zhang P., Feng K. A Clustering Fault Diagnosis Method for Gas Turbine Performance Parameters Based on t-SNE. Proceedings of the 2022 Global Reliability and Prognostics and Health Management (PHM-Yantai).

[3] Wu S., Russhard P., Yan R., Tian S., Wang S., Zhao Z., Chen X. (2020). An adaptive online blade health monitoring method: From raw data to parameters identification. IEEE Trans. Instrum. Meas..

[4] Feng K., Xiao Y., Li Z., Jiang Z., Gu F. (2023). Gas Turbine Blade Fracturing Fault Diagnosis Based on Broadband Casing Vibration. Measurement.

[5] Hu D., Zhang C., Yang T., Chen G. (2022). An Intelligent Anomaly Detection Method for Rotating Machinery Based on Vibration Vectors. IEEE Sens. J..

[6] Egaji O.A., Ekwevugbe T., Griffiths M. A Data Mining based Approach for Electric Motor Anomaly Detection Applied on Vibration Data. Proceedings of the 2020 Fourth World Conference on Smart Trends in Systems, Security and Sustainability (WorldS4).

[7] Wang H., Li Q., Liu Y., Yang S. (2022). Anomaly Data Detection of Rolling Element Bearings Vibration Signal Based on Parameter Optimization Isolation Forest. Machines.

[8] Yan W., Goebel K.F. (2003). Sensor validation and fusion for gas turbine vibration monitoring. Syst. Diag. Prog. Secur. Cond. Monit. III.

[9] Cao J., Dai H., Lei B., Yin C., Zeng H., Kummert A. (2020). Maximum correntropy criterion-based hierarchical one-class classification. IEEE Trans. Neural Netw. Learn Syst..

[10] Xu H., Song P., Liu B. A vibration signal anomaly detection method based on frequency component clustering and isolated forest algorithm. Proceedings of the 2019 IEEE 2nd International Conference on Automation, Electronics and Electrical Engineering (AUTEEE).

[11] Li J., Liu Y., Wang Q., Xing Z., Zeng F. (2022). Rotating machinery anomaly detection using data reconstruction generative adversarial networks with vibration energy analysis. AIP Adv..

[12] Yu J., Zhou X., Lu L., Zhao Z. (2021). Multiscale dynamic fusion global sparse network for gearbox fault diagnosis. IEEE Trans. Instrum. Meas..

[13] Kwon D., Natarajan K., Suh S.C., Kim H., Kim J. An Empirical Study on Network Anomaly Detection Using Convolutional Neural Networks. Proceedings of the 2018 IEEE 38th International Conference on Distributed Computing Systems.

[14] Wen G., Zhou H., Su Y., Chen X. (2023). Anomaly Detection Based on Conditional Variational Autoencoder for Bearing Operating Under Time Varying Conditions. J. Vib. Meas. Diag..

[15] Sun J., Yan C., Wen J. (2018). Intelligent Bearing Fault Diagnosis Method Combining Compressed Data Acquisition and Deep Learning. IEEE Trans. Instrum. Meas..

[16] Zhang S., Wang R., Si Y., Wang L. (2022). An Improved Convolutional Neural Network for Three-Phase Inverter Fault Diagnosis. IEEE Trans. Instrum. Meas..

[17] Li Z., Sun Y., Yang L., Zhao Z., Chen X. (2022). Unsupervised Machine Anomaly Detection Using Autoencoder and Temporal Convolutional Network. IEEE Trans. Instrum. Meas..

[18] Gao H., Qiu B., Barroso R.J.D., Hussain W., Xu Y., Wang X. (2023). TSMAE: A Novel Anomaly Detection Approach for Internet of Things Time Series Data Using Memory-Augmented Autoencoder. IEEE Trans. Netw. Sci. Eng..

[19] Ibrahim R., Zemouri R., Tahan A., Lafleur F., Kedjar B., Merkhouf A., Al-Haddad K. Anomaly Detection for Large Hydrogenerators Using the Variational Autoencoder Based on Vibration Signals. Proceedings of the 2022 International Conference on Electrical Machines (ICEM).

[20] Hoffmann J.L.C., Horstmann L.P., Lucena M.M., Araujo G.M., Frohlich A.A., Nishioka M.H.N. (2022). Anomaly Detection on Wind Turbines Based on a Deep Learning Analysis of Vibration Signals. Appl. Artif. Intell..

[21] Matsui A., Asahi S., Tamura S., Hayamizu S., Isashi R., Furukawa A., Naitou T. (2020). Anomaly Detection in Mechanical Vibration Using Combination of Signal Processing and Autoencoder. J. Signal Process..

[22] Liu S., Ji Z., Wang Y., Zhang Z., Xu Z., Kan C., Jin K. (2021). Multi-feature fusion for fault diagnosis of rotating machinery based on convolutional neural network. Comput. Commun..

[23] Chen Z., Li W. (2017). Multisensor Feature Fusion for Bearing Fault Diagnosis Using Sparse Autoencoder and Deep Belief Network. IEEE Trans. Instrum. Meas..

[24] Su H., Chong K.T. (2007). Induction Machine Condition Monitoring Using Neural Network Modeling. IEEE Trans. Ind. Electron..

[25] Kim S., Park H.J., Seo Y.H., Choi J.H. (2022). A Robust Health Indicator for Rotating Machinery Under Time-Varying Operating Conditions. IEEE Access.

[26] Schmidt S., Zimroz R., Heyns P.S. (2021). Enhancing gearbox vibration signals under time-varying operating conditions by combining a whitening procedure and a synchronous processing method. Mech. Syst. Signal Process..

[27] Schmidt S., Gryllias K.C. (2021). The anomalous and smoothed anomalous envelope spectra for rotating machine fault diagnosis. Mech. Syst. Signal Process..

[28] Schmidt S., Heyns P.S. (2020). Normalisation of the amplitude modulation caused by time-varying operating conditions for condition monitoring. Measurement.

[29] Chang S., Cheng M., Xu Y., Cheng L. (2019). Study on Foreign Object Damage Regular Pattern of Aero Engine Compressor Blades. J. Mech. Eng..

[30] Zhao X., Yao J., Deng W., Ding P., Ding Y., Jia M., Liu Z. (2022). Intelligent Fault Diagnosis of Gearbox Under Variable Working Conditions With Adaptive Intraclass and Interclass Convolutional Neural Network. IEEE Trans. Neural Netw. Learn Syst..

[31] Alzubaidi L., Zhang J., Humaidi A.J., Al-Dujaili A., Duan Y., Al-Shamma O., Santamaría J.I., Fadhel M.A., Al-Amidie M., Farhan L. (2021). Review of deep learning: Concepts, CNN architectures, challenges, applications, future directions. J. Big Data.

[32] Higgins I., Matthey L., Pal A., Burgess C.P., Glorot X., Botvinick M.M., Mohamed S., Lerchner A. beta-VAE: Learning Basic Visual Concepts with a Constrained Variational Framework. Proceedings of the 5th International Conference on Learning Representations.

[33] Burgess C.P., Higgins I., Pal A., Matthey L., Watters N., Desjardins G., Lerchner A. (2018). Understanding disentangling in *β*-VAE. arXiv.

[34] Yuan C., Yang H. (2019). Research on K-Value Selection Method of K-Means Clustering Algorithm. J.

[35] Tambunan H.B., Barus D.H., Hartono J., Alam A.S., Nugraha D.A., Usman H.H.H. Electrical Peak Load Clustering Analysis Using K-Means Algorithm and Silhouette Coefficient. Proceedings of the 2020 International Conference on Technology and Policy in Energy and Electric Power (ICT-PEP).

